# Supplementary material for: An Exploratory Bioinformatic Investigation of Cats’ Susceptibility to Coronavirus-Deriving Epitopes
Source: Life (Basel). 2024 Mar 2;14(3):334. doi: 10.3390/life14030334 (PMC10971392; doi:10.3390/life14030334)
Supplement: Supplementary file 1 [file life-14-00334-s001.zip › life-2868343-supplementary.pdf]

Table S1. Entries available on UniProt of alleles deriving from the three loci (E, H and K) of class-I FLA of Felis catus (Felis silvestris catus) selected for the studies. All the entries are marked as "unreviewed").

FLA-I E				FLA-I H				FLA-I K			
**Gene Names**	**Allele name**	**UniProt ID**	**Length**	**Gene Names**	**Allele name**	**UniProt ID**	**Length**	**Gene Names**	**Allele name**	**UniProt ID**	**Length**
FLAI-E	*00501	A0A516V164	362	FLAI-H	*00102	S4Z0I2	182	FLAI-K	*00401	S4Z0K5	275
FLAI-E	*00101	S4Z0J3	182	FLAI-H	*00601	S4Z0I6	182	FLAI-K	*00801	S4Z239	182
FLAI-E	*00701	S4Z0J6	182	FLAI-H	*003012	S4Z242	182	FLAI-K	*00701	S4Z3H3	275
FLAI-E	*01301	S4Z0K0	182	FLAI-H	*008011	S4Z246	182	FLAI-K	*00201	S4Z3H8	362
FLAI-E	*01401	S4Z0M6	275	FLAI-H	*00501	S4Z264	362	FLAI-K	*00101	S4Z3M6	182
FLAI-E	*01101	S4Z253	182	FLAI-H	*003011	S4Z3F6	182	FLAI-K	*00501	S4Z3N0	182
FLAI-E	*00303	S4Z3G3	182	FLAI-H	*00701	S4Z3F9	182	FLAI-K	*017	S4Z3R1	275
FLAI-E	*01001	S4Z3G5	182	FLAI-H	*00201	S4Z3N5	182	FLAI-K	*00303	S4Z9L2	182
FLAI-E	*00302	S4Z3P3	182	FLAI-H	*00401	S4Z3P0	182	FLAI-K	*00302	S4Z9P2	362
FLAI-E	*00902	S4Z3P7	182	FLAI-H	*00101	S4Z9L5	182				
FLAI-E	*00601	S4Z9M5	182	FLAI-H	*008012	S4Z9M3	182				
FLAI-E	*01201	S4Z9M8	182								

Table S2. Entries (marked as "reviewed") available on UniProt of glycoproteins deriving from SARS-CoV-2, FIPV and FeCV.

Severe acute respiratory syndrome coronavirus 2 (2019-nCoV) (SARS-CoV-2) Taxonomy ID: 2697049				
**Protein names**	**Entry Name**	**Gene Names**	**UniProt ID**	**Length**
Replicase polyprotein 1a (pp1a) (ORF1a polyprotein)	R1A_SARS2		P0DTC1	4405
Spike glycoprotein (S glycoprotein) (E2)	SPIKE_SARS2	S 2	P0DTC2	1273
ORF3a protein (ORF3a)	AP3A_SARS2	3a	P0DTC3	275
Envelope small membrane protein (E) (sM protein)	VEMP_SARS2	E 4	P0DTC4	75
Membrane protein (M) (E1 glycoprotein)	VME1_SARS2	M	P0DTC5	222
ORF6 protein (ORF6) (Non-structural protein 6) (ns6)	NS6_SARS2	6	P0DTC6	61
ORF7a protein (ORF7a) (Protein U122)	NS7A_SARS2	7a	P0DTC7	121
ORF8 protein (ORF8) (Non-structural protein 8) (ns8)	NS8_SARS2	8	P0DTC8	121
Nucleoprotein (N) (Nucleocapsid protein) (NC) (Protein N)	NCAP_SARS2	N	P0DTC9	419
Replicase polyprotein 1ab (pp1ab) (ORF1ab polyprotein)	R1AB_SARS2	rep 1a-1b	P0DTD1	7096
ORF9b protein (ORF9b) (ORF-9b) (Protein 9b)	ORF9B_SARS2	9b	P0DTD2	97
Putative ORF9c protein (ORF9c) (ORF14)	ORF9C_SARS2	9c	P0DTD3	73
ORF7b protein (ORF7b)	NS7B_SARS2	7b	P0DTD8	43
Putative ORF10 protein	ORF10_SARS2	ORF10	A0A663DJA2	38
Putative ORF3b protein (ORF3b)	ORF3B_SARS2		P0DTF1	22
Putative ORF3d protein	ORF3D_SARS2		P0DTG0	57
ORF3c protein (ORF3c)	ORF3C_SARS2		P0DTG1	41
**Feline coronavirus (strain FIPV WSU-79/1146) (FCoV) (Taxonomy ID: 33734)**				
**Protein names**	**Entry Name**	**Gene Names**	**Entry**	**Length**
Replicase polyprotein 1ab (pp1ab) (ORF1ab polyprotein)	R1AB_FIPV	rep 1a-1b	Q98VG9	6709
Spike glycoprotein (S glycoprotein) (E2)	SPIKE_FIPV	S 2	P10033	1452
Membrane protein (M protein) (E1 glycoprotein)	VME1_FIPV	M 5	P25878	262
Nucleoprotein (Nucleocapsid protein) (NC) (Protein N)	NCAP_FIPV	N 6	P25909	377

Non-structural protein 7a (ns7a)	NS7_FIPV	7a	P19742	101
Envelope small membrane protein (E protein) (sM protein)	VEMP_FIPV	E sM	Q52PA5	82
Non-structural protein 3a (ns3a)	NS3A_FIPV	3a	O39432	71
Non-structural protein 3x (ns3x)	NS3X_FIPV	3x	O39433	71
Putative truncated non-structural protein 3b (ns3b)	NS3B_FIPV	3b	P0C545	40
Non-structural protein 7b (ns7b)	NS7B_FIPV	7b	P19743	206
**Feline enteric coronavirus (strain 79-1683) (FeCoV) (FeCV) (Taxonomy ID: 33733)**				
**Protein names**	**Entry Name**	**Gene Names**	**Entry**	**Length**
Non-structural 7a protein (ns7a)	NS7_CVFE3	7a	P33465	101
Non-structural protein 7b (ns7b)	NS7B_CVFE3	7b	P33467	176

Table S3. Number of epitopes deriving from viral glycoproteins predicted by NetMHCPan with EL score > 0.5 and > 0.8 for each allele in FLA-I E locus.

	Organism	FeCV		FIPV												SARS-CoV-2											Total epitopes
	Protein	NS7B	NS7C	NS3A	NS3X	NS3B	SPIKE	NS7F	NS7B	VME1	NCAP	VEMP	R1AB	R1A	SPIKE	AP3A	VEMP	VME1	NS6	NS7A	NS8	NCAP	R1AB	ORF9B	
FLA-I E*00501	EL > 0.5	1	-	-	-	-	4	-	1	1	1	2	22	18	7	1	-	1	-	1	-	3	26	-	89
	EL > 0.8	-	-	-	-	-	-	-	-	-	-	-	1	-	-	-	-	-	-	-	-	-	-	-	1
FLA-I E*00101	EL > 0.5	3	-	1	1	-	23	-	1	3	4	2	89	67	21	4	1	2	-	-	-	6	102	2	332
	EL > 0.8	-	-	-	-	-	1	-	-	1	1	-	10	10	3	-	-	-	-	1	-	2	12	-	41
FLA-I E*00701	EL > 0.5	1	-	-	-	-	5	-	-	1	2	1	23	18	7	2	-	1	-	1	-	2	29	-	93
	EL > 0.8	-	-	-	-	-	-	-	-	-	-	-	2	1	-	-	-	-	-	-	-	-	1	-	4
FLA-I E*01301	EL > 0.5	1	-	1	1	-	7	-	1	3	1	2	37	33	7	1	1	3	-	1	-	4	52	3	159
	EL > 0.8	-	-	-	-	-	-	-	-	-	-	-	-	-	-	-	-	-	-	-	-	-	1	-	1
FLA-I E*01401	EL > 0.5	-	-	1	-	-	-	-	-	-	-	-	8	6	-	-	-	-	-	1	-	-	14	1	31
	EL > 0.8	-	-	-	-	-	-	-	-	-	-	-	-	-	-	-	-	-	-	-	-	-	-	-	0
FLA-I E*01101	EL > 0.5	1	1	-	2	-	19	1	2	-	5	1	83	51	19	8	1	2	1	2	1	5	95	3	303
	EL > 0.8	-	-	-	-	-	-	-	-	-	-	-	4	2	-	1	-	-	-	-	-	-	3	1	11
FLA-I E*00303	EL > 0.5	1	-	-	-	-	1	-	-	-	-	-	8	7	3	-	-	-	-	1	-	2	8	-	31
	EL > 0.8	-	-	-	-	-	-	-	-	-	-	-	-	-	-	-	-	-	-	-	-	-	-	-	0
FLA-I E*01001	EL > 0.5	2	-	-	-	-	16	-	1	2	4	3	41	42	7	5	-	3	-	1	-	4	72	3	206
	EL > 0.8	-	-	-	-	-	-	-	-	-	-	-	2	-	-	-	-	-	-	-	-	-	-	1	3
FLA-I E*00302	EL > 0.5	1	-	-	-	-	1	-	-	-	-	-	8	7	3	-	-	-	-	1	-	2	8	-	31
	EL > 0.8	-	-	-	-	-	-	-	-	-	-	-	-	-	-	-	-	-	-	-	-	-	-	-	0
FLA-I E*00902	EL > 0.5	1	-	-	1	-	8	-	-	2	2	1	27	25	5	3	-	2	-	2	-	3	44	3	129
	EL > 0.8	-	-	-	-	-	-	-	-	-	-	-	-	1	-	-	-	-	-	-	-	-	1	1	3
FLA-I E*00601	EL > 0.5	1	-	-	-	-	1	-	-	-	-	-	2	4	-	-	-	-	-	-	-	-	4	-	12
	EL > 0.8	-	-	-	-	-	-	-	-	-	-	-	-	-	-	-	-	-	-	-	-	-	-	-	0
FLA-I E*01201	EL > 0.5	-	-	1	-	-	1	-	2	1	1	-	20	18	4	2	-	-	-	-	-	2	28	1	81
	EL > 0.8	-	-	-	-	-	-	-	-	-	-	-	-	3	-	-	-	-	-	-	-	1	4	-	8

Table S4. Number of epitopes deriving from viral glycoproteins predicted by NetMHCPan with EL score > 0.5 and > 0.8 for each allele in FLA-I H locus.

	Organism	FeCV		FIPV												SARS-CoV-2											Total epitopes
	Protein	NS7B	NS7C	NS3A	NS3X	NS3B	SPIKE	NS7F	NS7B	VME1	NCAP	VEMP	R1AB	R1A	SPIKE	AP3A	VEMP	VME1	NS6	NS7A	NS8	NCAP	R1AB	ORF9B	
FLA-I H*00102	EL > 0.5	3	-	-	1	-	22	-	2	7	1	3	72	55	17	4	-	3	-	1	1	3	89	1	285
	EL > 0.8	-	-	-	-	-	3	-	-	1	-	-	2	1	1	1	-	-	-	-	-	-	3	-	12
FLA-I H*00601	EL > 0.5	2	-	-	1	-	16	-	1	4	2	2	53	35	12	2	-	4	-	2	-	3	66	-	205
	EL > 0.8	-	-	-	-	-	1	-	-	-	-	-	-	1	-	-	-	-	-	-	-	-	1	-	3
FLA-I H*003011	EL > 0.5	2	-	-	-	-	18	-	3	6	2	2	44	35	8	6	1	3	-	1	1	3	66	1	202
	EL > 0.8	-	-	-	-	-	1	-	-	-	-	-	-	-	-	-	-	-	-	-	-	-	2	-	3

**FLA-I H*00701**	EL > 0.5	4	-	-	1	-	30	-	1	9	6	5	77	60	12	7	2	5	-	1	1	4	106	1	332
	EL > 0.8	-	-	-	-	-	3	-	-	1	2	1	7	6	2	1	-	-	-	-	-	2	12	-	37
**FLA-I H*00501**	EL > 0.5	3	-	-	1	-	25	-	1	6	5	4	70	52	13	7	1	4	-	1	2	4	89	1	289
	EL > 0.8	-	-	-	-	-	-	-	-	-	1	-	1	1	-	-	-	-	-	-	-	-	4	-	7
**FLA-I H*00401**	EL > 0.5	5	-	-	2	-	36	-	2	10	6	5	98	70	15	9	2	8	-	2	1	5	125	1	402
	EL > 0.8	-	-	-	-	-	4	-	-	1	3	-	9	9	2	1	-	-	-	-	-	2	15	-	46
**FLA-I H*008012**	EL > 0.5	2	-	1	1	-	37	-	1	14	6	4	126	92	24	8	3	8	-	2	1	6	157	1	494
	EL > 0.8	-	-	-	-	-	5	-	-	1	1	1	15	6	3	1	-	-	-	-	-	-	13	-	46
**FLA-I H*003012**	EL > 0.5	2	-	-	-	-	18	-	3	6	2	2	44	35	8	6	1	3	-	1	1	3	66	1	202
	EL > 0.8	-	-	-	-	-	1	-	-	-	-	-	-	-	-	-	-	-	-	-	-	-	2	-	3
**FLA-I H*008011**	EL > 0.5	2	-	1	1	-	37	-	1	14	6	4	126	92	24	8	3	8	-	2	1	6	157	1	494
	EL > 0.8	-	-	-	-	-	5	-	-	1	1	1	15	6	3	1	-	-	-	-	-	-	13	-	46
**FLA-I H*00201**	EL > 0.5	2	-	1	-	-	23	-	1	6	3	3	67	46	14	6	-	3	-	1	-	2	87	1	266
	EL > 0.8	-	-	-	-	-	-	-	-	-	-	-	-	1	2	1	-	-	-	-	-	-	1	-	5
**FLA-I H*00101**	EL > 0.5	3	-	-	1	-	22	-	2	7	1	3	72	55	17	4	-	3	-	1	1	3	89	1	285
	EL > 0.8	-	-	-	-	-	3	-	-	1	-	-	2	1	1	1	-	-	-	-	-	-	3	-	12

Table S5. Number of epitopes deriving from viral glycoproteins predicted by NetMHCPan with EL score > 0.5 and > 0.8 for each allele in FLA-I K locus.

	**Organism**	**FeCV**		**FIPV**										**SARS-CoV-2**											
	**Protein**	**NS7B**	**NS7C**	**NS3A**	**NS3X**	**NS3B**	**SPIKE**	**NS7F**	**NS7B**	**VME1**	**NCAP**	**VEMP**	**R1AB**	**R1A**	**SPIKE**	**AP3A**	**VEMP**	**VME1**	**NS6**	**NS7A**	**NS8**	**NCAP**	**R1AB**	**ORF9B**	**Total epitopes**
**FLA-I K*00401**	EL > 0.5	1	-	-	-	-	2	-	-	-	-	-	5	2	-	1	-	-	-	-	-	1	6	1	19
	EL > 0.8	-	-	-	-	-	-	-	-	-	-	-	-	-	-	-	-	-	-	-	-	-	-	-	0
**FLA-I K*00302**	EL > 0.5	1	-	-	-	-	1	-	2	1	1	-	9	4	-	1	-	-	-	-	-	2	10	1	33
	EL > 0.8	-	-	-	-	-	-	-	-	-	-	-	-	-	-	-	-	-	-	-	-	-	-	-	0
**FLA-I K*00801**	EL > 0.5	4	-	-	3	-	16	-	2	5	6	2	79	57	16	4	1	1	-	2	1	4	103	4	310
	EL > 0.8	1	-	-	2	-	1	-	1	1	1	-	11	8	2	-	-	1	-	-	-	1	19	-	49
**FLA-I K*00201**	EL > 0.5	-	-	-	-	-	-	-	-	-	-	-	-	-	-	-	-	-	-	-	-	-	-	-	0
	EL > 0.8	-	-	-	-	-	-	-	-	-	-	-	-	-	-	-	-	-	-	-	-	-	-	-	0
**FLA-I K*00701**	EL > 0.5	1	-	-	2	-	4	-	2	1	3	-	46	24	11	4	-	1	-	1	-	5	48	2	155
	EL > 0.8	-	-	-	-	-	-	-	-	-	1	-	-	-	-	-	-	-	-	-	-	-	1	-	2
**FLA-I K*00101**	EL > 0.5	-	-	-	-	-	-	-	-	-	-	-	-	-	-	-	-	-	-	-	-	-	-	-	0
	EL > 0.8	-	-	-	-	-	-	-	-	-	-	-	-	-	-	-	-	-	-	-	-	-	-	-	0
**FLA-I K*00501**	EL > 0.5	1	-	-	-	-	5	-	1	1	-	1	15	11	1	2	-	-	-	-	-	1	17	1	57
	EL > 0.8	-	-	-	-	-	-	-	-	-	-	-	-	-	-	-	-	-	-	-	-	-	-	-	0
**FLA-I K*00303**	EL > 0.5	1	-	-	-	-	1	-	2	1	1	-	9	4	-	1	-	-	-	-	-	2	10	1	33
	EL > 0.8	-	-	-	-	-	-	-	-	-	-	-	-	-	-	-	-	-	-	-	-	-	-	-	0
**FLA-I K*017**	EL > 0.5	1	-	-	-	-	14	-	2	1	6	3	60	53	11	4	-	1	-	2	-	3	81	3	245
	EL > 0.8	-	-	-	-	-	-	-	-	-	-	-	2	1	-	-	-	-	-	-	-	-	3	1	7

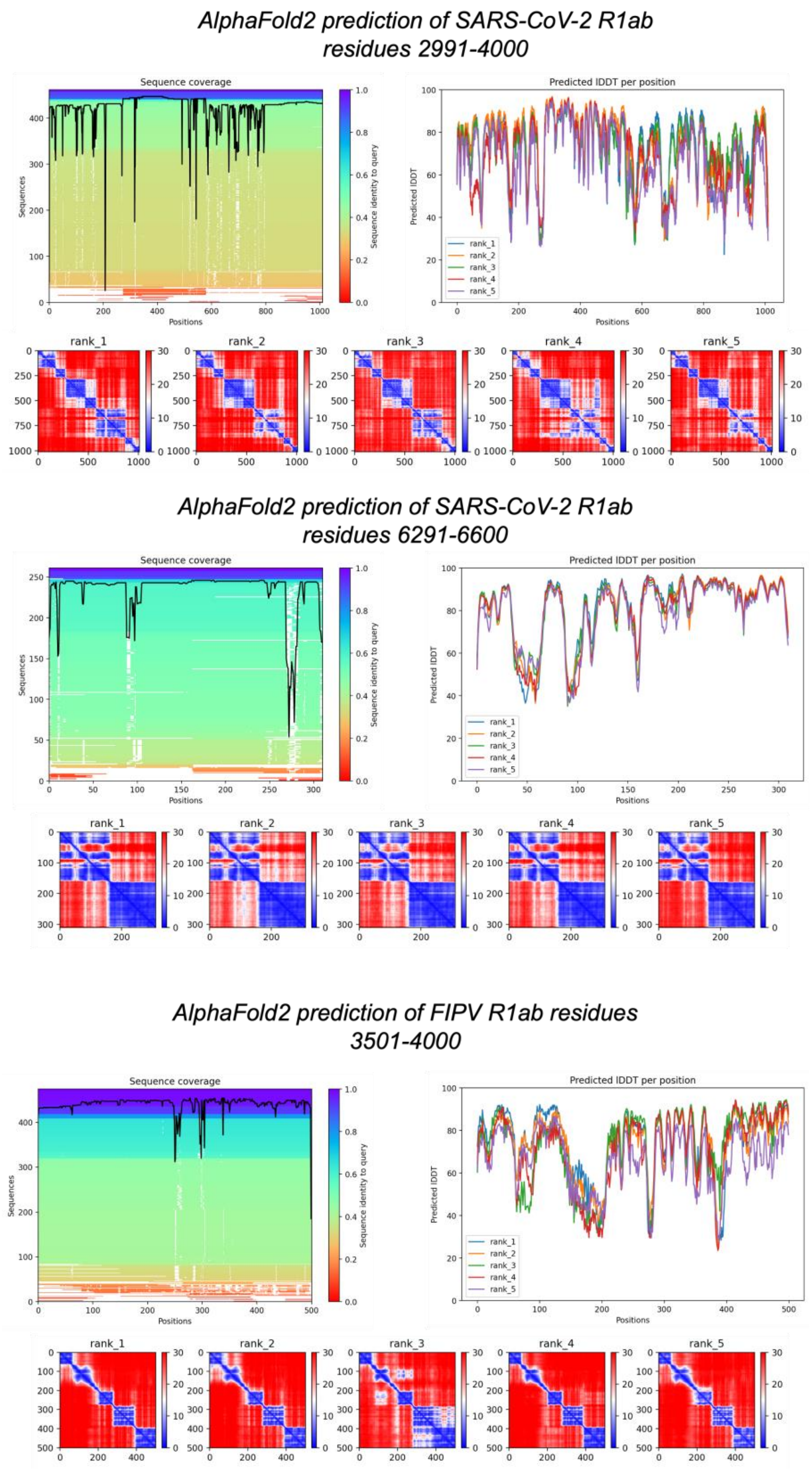

Figure S1. Results of the AlphaFold2 prediction through ColabFold server of the two moieties of SARS-CoV-2 R1ab between the residues 2991-4000 and 6291-6600 and FIPV R1ab residues 3501-4000. The graphs generated by ColabFold report **A)** the coverage of the sequences derived from the multiple sequence alignment (MSA) in UniRef100 server, **B)** the predicted local distance difference test (pLDDT) score for the 5 models generated and **C)** the

predicted alignment error (PAE), indicating a distance score between pair of residues with low values specifying low errors.

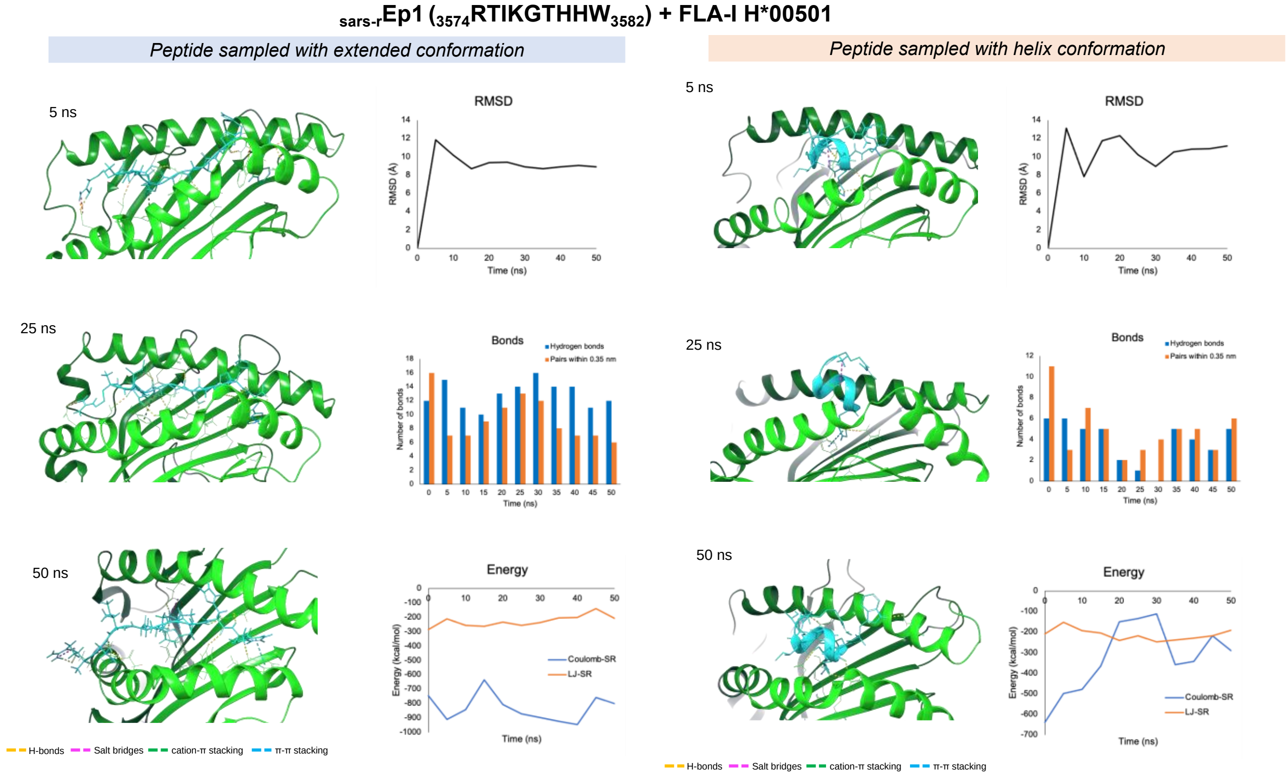

Figure S2. Results of the 50 ns classical MD simulations of $_{sars\text{-}r}$Ep1 ($_{3574}$RTIKGTHHW$_{3582}$) in complex with FLA-I H*00501. The peptide was sampled in two poses (extended and helix), as derived from molecular docking results. The screenshots were taken at 5, 25 and 50 ns. The plots report from the top to the bottom: RMSD of atom position in protein backbones with respect to the system as a function of time; number of H-bonds (blue bars) and contacts within 0.35 nm (orange bars) established in the MD as a function of time; short-range Coulomb (blue line) and Lennard-Jones (orange line) energies calculated for each timestep of the MD.

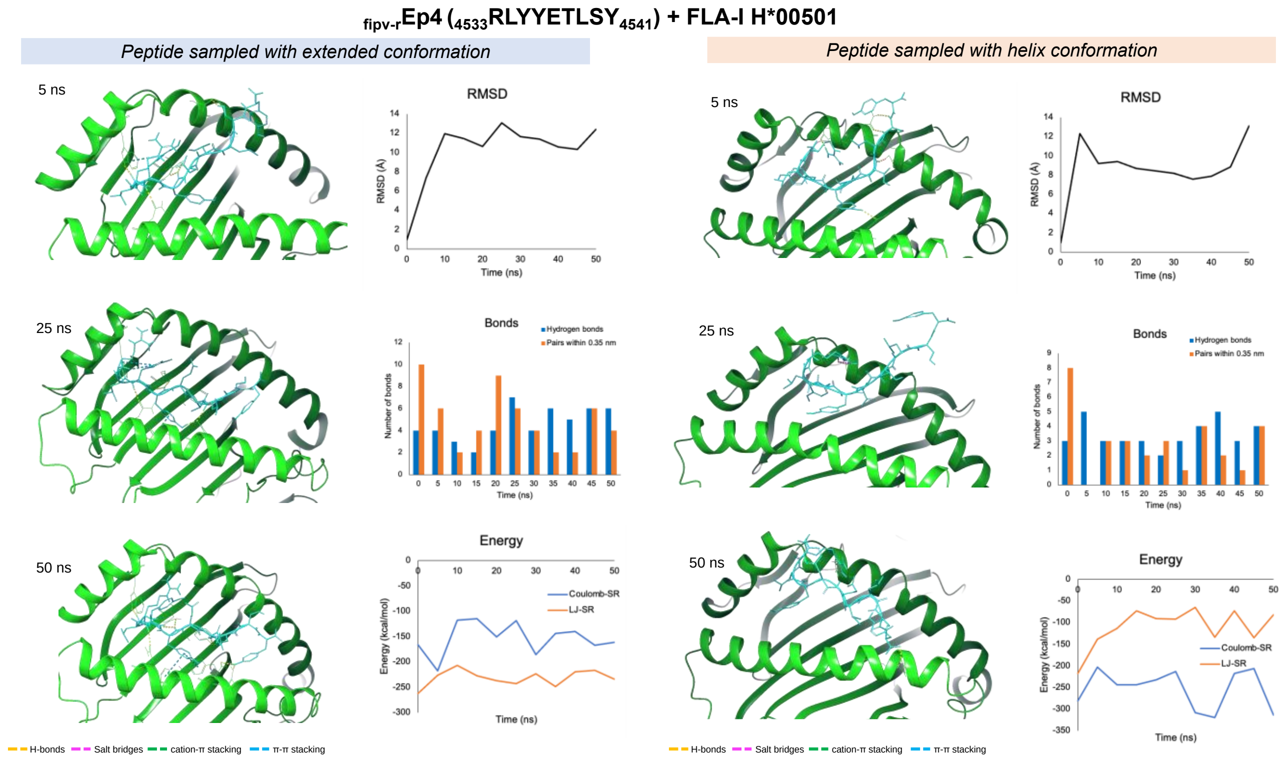

Figure S3. Results of the 50 ns classical MD simulations of $_{fipv\text{-}r}$Ep4 ($_{4533}$RLYYETLSY$_{4541}$) in complex with FLA-I H*00501. The peptide was sampled in two poses (extended and helix), as derived from molecular docking results. The screenshots were taken at 5, 25 and 50 ns. The plots report from the top to the bottom: RMSD of atom position in protein backbones with respect to the system as a function of time; number of H-bonds (blue bars) and contacts within 0.35 nm (orange bars) established in the MD as a function of time; short-range Coulomb (blue line) and Lennard-Jones (orange line) energies calculated for each timestep of the MD.

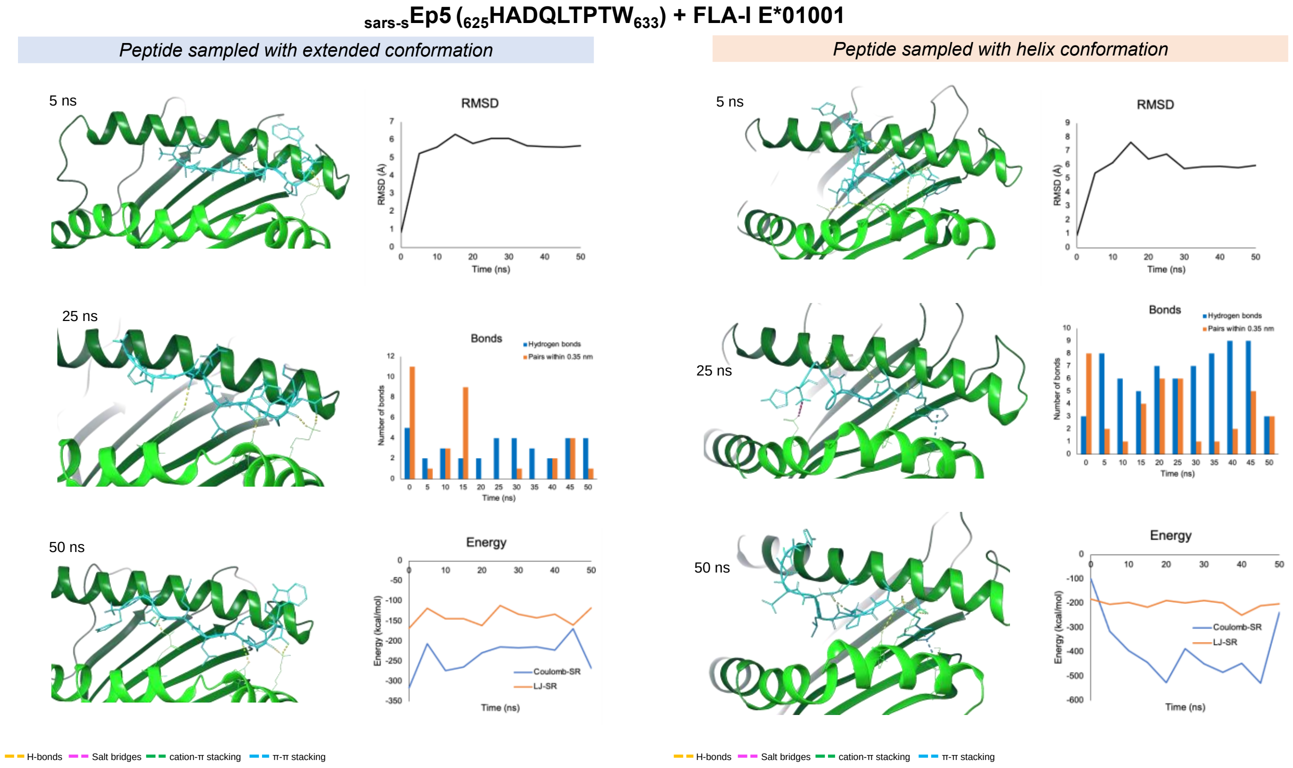

Figure S4. Results of the 50 ns classical MD simulations of $_{\text{sars-s}}$Ep5 ($_{625}$HADQLTPTW$_{633}$) in complex with FLA-I E*01001. The peptide was sampled in two poses (extended and helix), as derived from molecular docking results. The screenshots were taken at 5, 25 and 50 ns. The plots report from the top to the bottom: RMSD of atom position in protein backbones with respect to the system as a function of time; number of H-bonds (blue bars) and contacts within 0.35 nm (orange bars) established in the MD as a function of time; short-range Coulomb (blue line) and Lennard-Jones (orange line) energies calculated for each timestep of the MD.

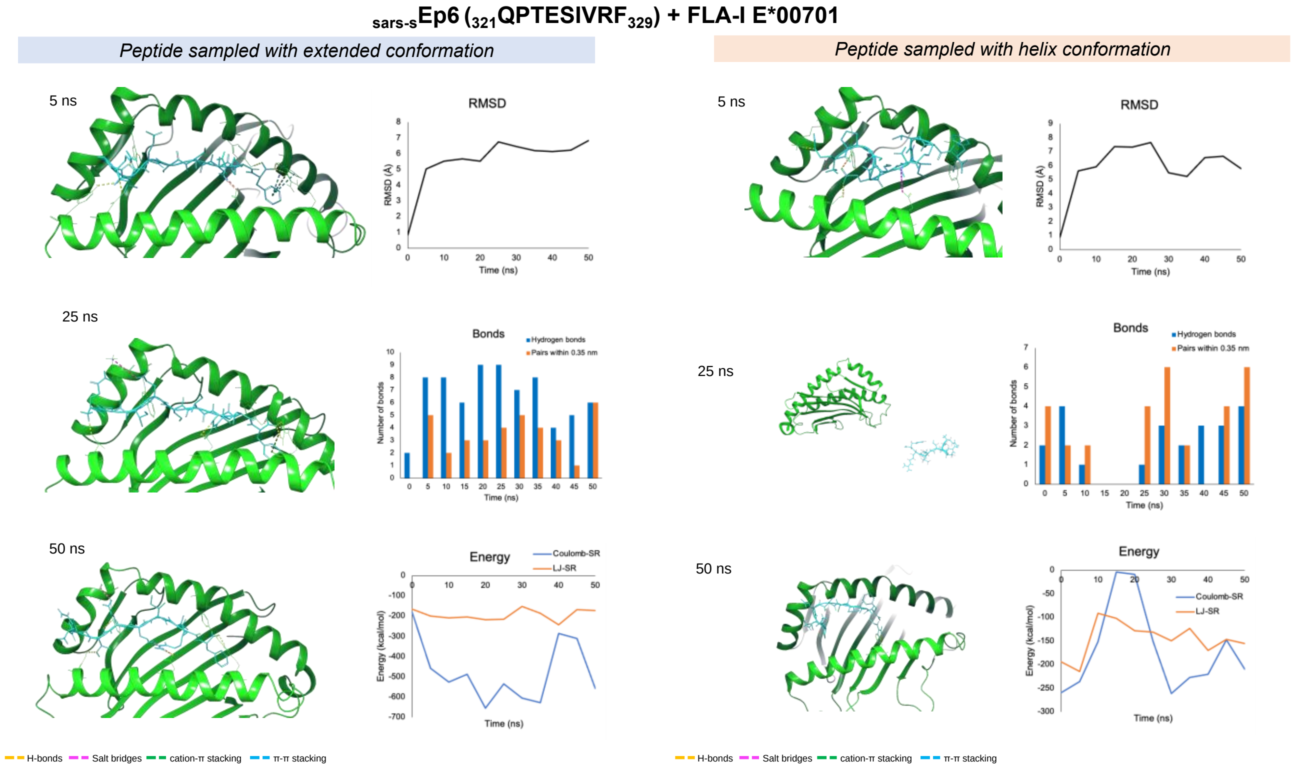

Figure S5. Results of the 50 ns classical MD simulations of $_{\text{sars-s}}$Ep6 ($_{321}$QPTESIVRF$_{329}$) in complex with FLA-I E*00701. The peptide was sampled in two poses (extended and helix), as derived from molecular docking results. The screenshots were taken at 5, 25 and 50 ns. The plots report from the top to the bottom: RMSD of atom position in protein backbones with respect to the system as a function of time; number of H-bonds (blue bars) and contacts within 0.35 nm (orange bars) established in the MD as a function of time; short-range Coulomb (blue line) and Lennard-Jones (orange line) energies calculated for each timestep of the MD.

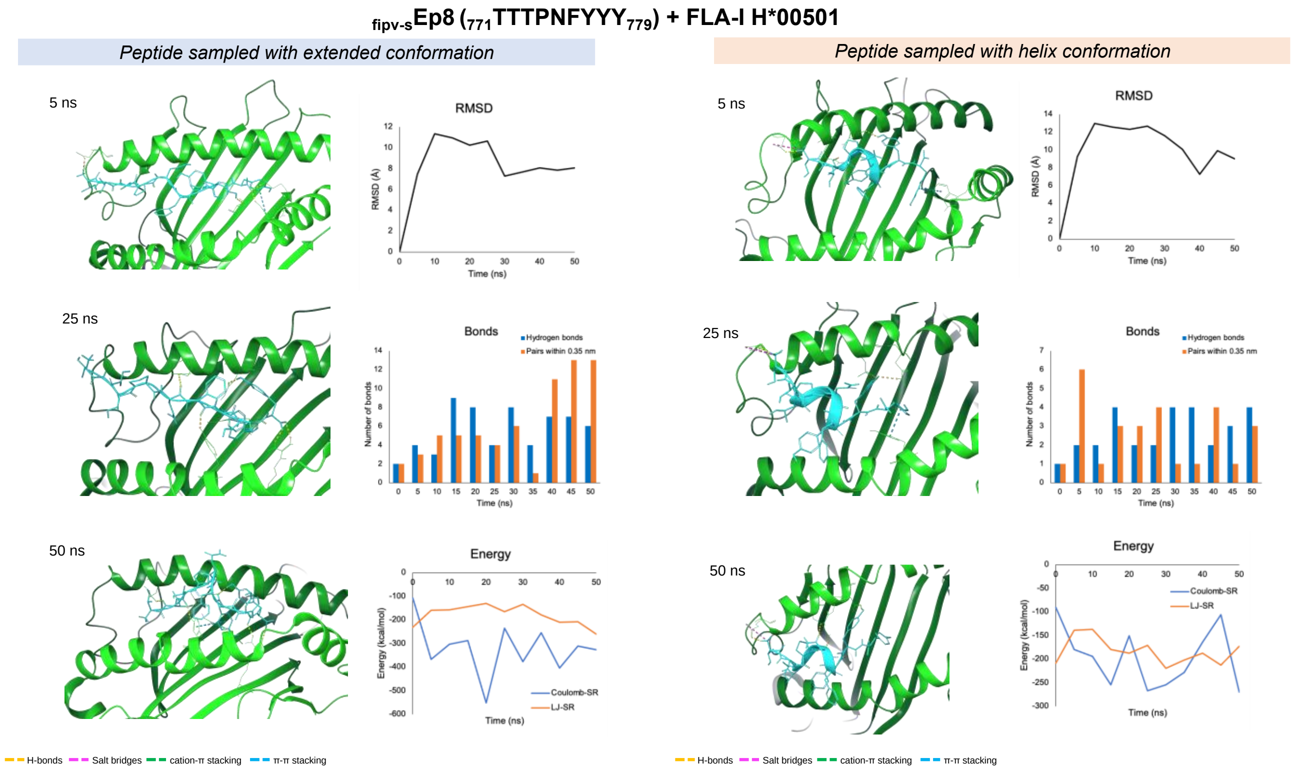

Figure S6. Results of the 50 ns classical MD simulations of $_{\text{fipv-s}}$Ep8 ($_{771}$TTTPNFYYY$_{779}$) in complex with FLA-I H*00501. The peptide was sampled in two poses (extended and helix), as derived from molecular docking results. The screenshots were taken at 5, 25 and 50 ns. The plots report from the top to the bottom: RMSD of atom position in protein backbones with respect to the system as a function of time; number of H-bonds (blue bars) and contacts within 0.35 nm (orange bars) established in the MD as a function of time; short-range Coulomb (blue line) and Lennard-Jones (orange line) energies calculated for each timestep of the MD.

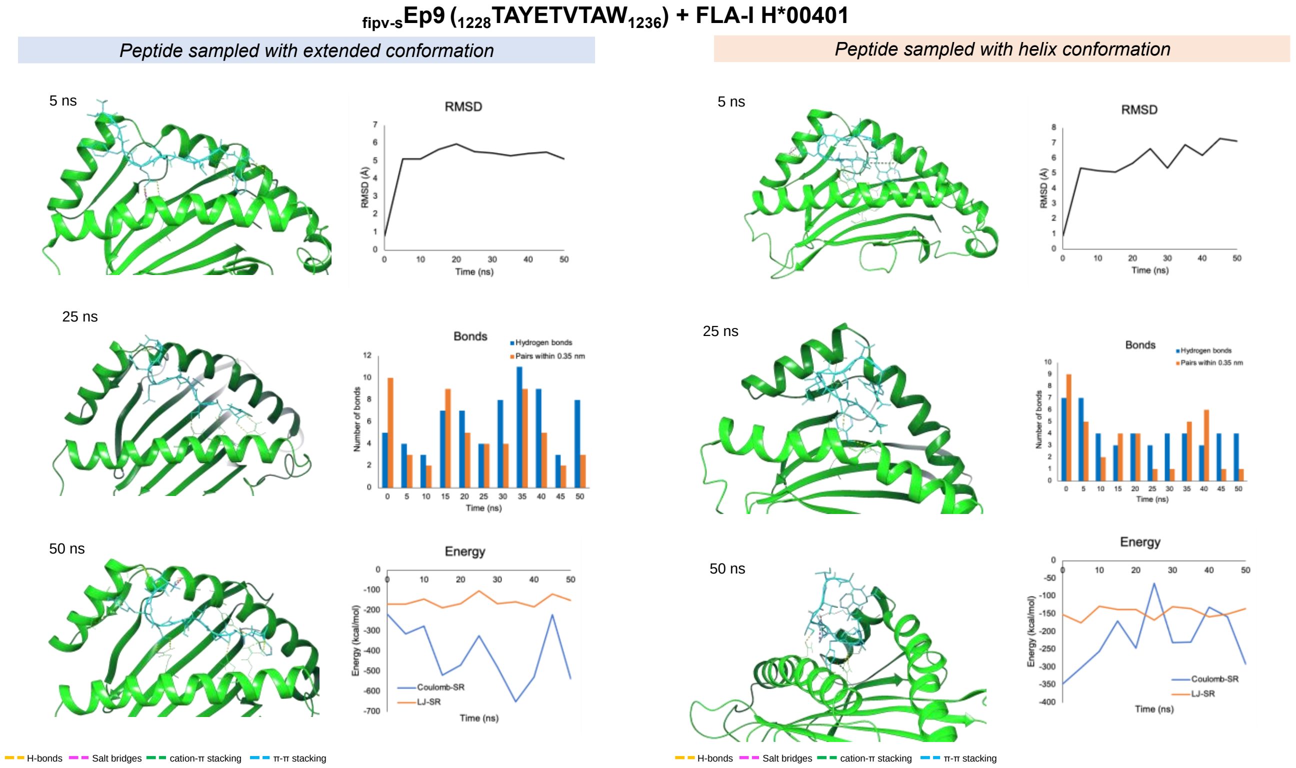

Figure S7. Results of the 50 ns classical MD simulations of $_{\text{fipv-s}}$Ep9 ($_{1228}$TAYETVTAW$_{1236}$) in complex with FLA-I H*00401. The peptide was sampled in two poses (extended and helix), as derived from molecular docking results. The screenshots were taken at 5, 25 and 50 ns. The plots report from the top to the bottom: RMSD of atom position in protein backbones with respect to the system as a function of time; number of H-bonds (blue bars) and contacts within 0.35 nm (orange bars) established in the MD as a function of time; short-range Coulomb (blue line) and Lennard-Jones (orange line) energies calculated for each timestep of the MD.
